# Extreme heat and cognition among older Mexican American adults

**DOI:** 10.1002/alz70860_099810

**Published:** 2025-12-23

**Authors:** Sadaf Arefi Milani, Izabella Galindo, Alexandra Holland, Phillip A Cantu, Kyriakos S. Markides, Brian Downer, Mukaila A Raji

**Affiliations:** ^1^ University of Texas Medical Branch, Galveston, TX, USA; ^2^ UTMB, Galveston, TX, USA

## Abstract

**Background:**

As extreme heat events become increasingly common, it is crucial to understand their health effects on older adults, who are particularly vulnerable to adverse outcomes associated with heat exposure. One area of concern is cognition, which may be affected directly, through heat‐related physical health deterioration, and indirectly, via factors such as social isolation and depression. Our objective is to assess the association between extreme heat and cognition among older Mexican American adults

**Method:**

We used data from the Hispanic Established Population for the Epidemiologic Study of the Elderly (HEPESE; 2004/2005‐ 2015/2016), a longitudinal study of older Mexican American adults living in the Southwestern US. We limited this analysis to participants residing in Texas or California. Heat data was sourced from the GridMET database, which was linked to the HEPESE through the Contextual Data Resource at the University of Southern California. We used daily maximum heat index values to create a yearly count of days with 1) a maximum heat index of 90°F+ (extreme heat) and 2) a maximum heat index of 104+ (dangerous heat). Cognition was measured at each wave with the Mini‐Mental State Examination (MMSE). We used mixed effects models to assess the longitudinal association between yearly count of extreme/dangerous heat days and MMSE score, adjusting for demographic and health covariates.

**Result:**

Our sample (*n* = 1,723) had an average age of 81.7 [standard deviation (SD): 5.1] and was 61.5% female. At baseline, the average MMSE score was 20.8 (SD: 7.2). On average, participants were exposed to 96.6 extreme heat days (SD: 60.6) and 18.1 dangerous heat days (SD: 21.6) at baseline. Extreme heat was associated with worse cognition at baseline [β: ‐0.008; 95% confidence interval (CI): ‐0.016, ‐0.001] and with decline over time (β: ‐0.003; 95% CI: ‐0.004, ‐0.002). Similarly, dangerous heat was associated with worse cognition at baseline (β: ‐0.033; 95% CI: ‐0.050, ‐0.017) and decline over time (β: ‐0.005; 95% CI: ‐0.007, ‐0.003).

**Conclusion:**

We found that daily heat indices greater than 90°F were associated with worse cognition over time among older Mexican Americans. Future work is needed to understand the pathways through which heat influences cognition.

